# Porcine deltacoronavirus infection triggers mitophagy to dampen the interferon response and promote viral replication

**DOI:** 10.3389/fimmu.2025.1684178

**Published:** 2025-10-13

**Authors:** Yue Chen, Yuan Zhao, Qingqing Song, Shijing Zhang, Zhenbang Zhu, Wenqiang Wang, Wei Wen, Xiangdong Li

**Affiliations:** ^1^ Jiangsu Co-innovation Center for Prevention and Control of Important Animal Infectious Diseases and Zoonoses, College of Veterinary Medicine, Yangzhou University, Yangzhou, China; ^2^ SINDER Animal Health, Zhucheng, China; ^3^ Joint International Research Laboratory of Agriculture and Agri-Product Safety, The Ministry of Education of China, Yangzhou University, Yangzhou, China; ^4^ Key Laboratory of Protection and Utilization of Biological Resources in Tarim Basin, College of Life Sciences, Tarim University, Alar, China

**Keywords:** porcine deltacoronavirus (PDCoV), mitophagy, MAVS, interferons production inhibition, mitochondrial damage

## Abstract

Porcine deltacoronavirus (PDCoV) is a newly emerging enteric pathogenic that causes severe diarrhea in neonatal piglets worldwide and presents a significant public health threat due to its potential for cross-species transmission. MAVS (Mitochondrial Antiviral Signaling Protein), serves as a crucial immune hub that connects virus recognition (RIG-I/MDA5) and interferon response. In this study, we found that PDCoV infection damage mitochondrial structure and function, as shown by mitochondrial membrane potential depolarization and reduction in mitochondrial numbers. In addition, PDCoV infection triggered mitophagy to eliminate the impaired mitochondria and degradation of MAVS, which resulted in a suppression of Interferon type I (IFN-I) production, thereby promoting viral replication. In conclusion, the data of this study indicate that PDCoV can degrade MAVS through mitophagy to weak the production of IFN-I, thereby promoting virus replication.

## 1 Introduction

Porcine deltacoronavirus (PDCoV) is a newly emerging enteric pathogenic coronavirus that infects piglets, which belongs to the genus Deltacoronavirus of the family Coronaviridae (1, 2). Similar to other swine enteric coronavirus, such as porcine epidemic diarrhea virus (PEDV) and porcine transmissible gastroenteritis virus (TGEV), PDCoV can cause acute diarrhea, vomiting, dehydration and mortality in nursing pigs (3, 4). PDCoV also cause a significance public concern as the capability of cross-species transmission and infect multiple species (4, 5). A study has reported that PDCoV can infect humans and cause systemic viral infection in children in Haiti (6). The PDCoV genome is a positive sense single-stranded RNA of approximately 25.4 kb in length that has an open reading frames 1a (ORF1a) and 1b (ORF1b) at the 5’ end of the genome, which including 15 mature nonstructural proteins (NSPs) processed by two polyprotein precursors: papain-like protease (PLpro, NSP3) and the 3C-like protease (NSP5) (7, 8). It also encodes four structural proteins, namely spike (S), envelope (E), membrane (M), and nucleocapsid (N) proteins (9).

Mitochondria are multifunctional organelles involved in energy, metabolism, signaling and death regulation. Their dynamic network (fusion/fission) and interactions with endoplasmic reticulum or other organelles further expand their functional dimensions (10–13). An increasing number of studies have shown that viral infections can damage morphology and function of mitochondria. For instance, the inner membrane and cristae of the mitochondria become deformed or even disappear after pseudorabies virus (PRV) infects PK-15 cells (14). PDCoV infection in LLC-PK1 cells also caused mitochondrial cristae deformed (3).

Autophagy is the major intracellular degradation system by which cytoplasmic materials are delivered to and degraded in the lysosome, which can be divided in to non-selective and selective in cell quality (10, 15, 16). Recent studies show that damaged mitochondria can be eliminated by selective autophagy for maintaining Adenosine Triphosphate (ATP) synthesis, reducing the level of Reactive Oxygen Species (ROS) and promoting virus replication (13). Some studies indicate that viruses can utilize mitophagy to inhibit the production of IFN-I, highlighting the crucial role of mitochondria in immunity, including the generation of antiviral IFN-I responses (14, 17). Mitochondria antiviral signaling protein (MAVS), located in the outer mitochondrial membrane (OMM), plays a crucial role in IFN-I production mediated by retinoic acid-inducible gene I-like receptors (RIG-I) and melanoma differentiation-associated protein 5 (MDA5). Coronavirus RNA can be recognized by RIG-I and/or MDA5 in the cytoplasm (18, 19), then MAVS is activated by upstream signaling from RIG-I and MDA5. Subsequently, interferon regulatory factor 3 (IRF3) and nuclear factor kappa-light-chain-enhancer of activated B cells are phosphorylated and translocated into nucleus to promote the production of interferons (IFNs), IFN-stimulated genes (ISGs), and inflammatory cytokines (14). Li et al. demonstrated that ORF10 of SARS-CoV-2 can suppress the antiviral innate response by degrading MAVS through mitophagy (20). However, whether PDCoV induce mitophagy during viral replication remains unclear.

In this study, we demonstrate that PDCoV infection utilizes mitophagy to inhibit IFN-I responses to promote virus replication. Mitophagy is triggered by PDCoV-induced damage of mitochondria and is associated with the degradation of MAVS, thereby suppress the host antiviral response.

## 2 Materials and methods

### 2.1 Chemical reagents

Reagents used were as follows: Enhanced Mitochondrial Membrane Potential Assay Kit with JC-1 (Beyotime, C2003S), Cell Mitochondria Isolation Kit (Beyotime, C3601), Reactive Oxygen Species Assay Kit (Beyotime, S0033S), phenylmethanesulfonyl fluoride (PMSF) (Beyotime, ST506), chloroquine (CQ, MCE, HY-17589A), MG132 (Selleck, S2619), Mdivi-1 (Beyotime, SC8028), PEI40000 (YESEN, 40816ES01), carbonyl cyanide3-chlorophenyl-hydrazone (CCCP, MCE, 555-60-2), rapamycin (Rapa, MCE, 53123-88-9).

Antibodies used were as follows: Tom20 (Santa Cruz, sc-17764), Lamp1(CST, 9091), COX- 4 (CST, 4850), GAPDH (CST, 5174), SQSTM1/P62 (CST, 8025), LC3B (CST, 3868), MAVS (Santa, sc-365333), β-actin (CST, 4970), FUNDC1 (Invitrogen, PA5-58535), Parkin (CST, 4211), TOLLIP (CST, 4748), PDCoV-S (house-made and preserved in our laboratory).

### 2.2 Cells and viruses

LLC-PK1 (porcine kidney epithelial line) cells were generous gift from Prof. Yaowei Huang from South China Agricultural University, and cultured in Dulbecco’s modified Eagle medium (HyClone, SH30243.01) containing 10% fetal bovine serum (Lonsera, S711-001S) and 100 U/mL penicillin/0.1 mg/mL streptomycin at 37 °C with 5% CO_2_. PEI40000 Transfection Reagent was employed for all transfection assays adhering to the manufacturer’s protocol. PDCoV stock was also provided by Prof. Yaowei Huang from South China Agricultura University, and proliferated on LLC-PK1 cells containing 5µg/mL Trypsin (Gibco: 25200-054) and 0.03% Tryptose Phosphate Broth (Sigma: T8159) at 37 °C with 5% CO_2_.

### 2.3 Flow cytometry

To assess the mitochondrial membrane potential (MMP), cells were incubated with 10 µM JC-1 probe for 20 min at 37 °C after virus infection. Cells were collected to detect the fluorescence intensity by flow cytometry (Becton-Dickinson, LSRFortessa). In healthy mitochondria with high MMP, JC-1 is incorporated in high concentration and forms aggregates that emit red fluorescence. In damaged mitochondria that with low MMP, JC-1 is de-aggregated into monomers that emit green fluorescence. The ratio of red fluorescence to green fluorescence was calculated to indicate the level of MMP. To assess ROS levels, cells were incubated with 10 µM DCFH-DA dye for 20 min at 37 °C after virus infection (14). Cells were collected to detect the fluorescence intensity by flow cytometry. The mean fluorescence intensity of cells was analyzed using the FlowJo V10 (TreeStar, USA). Three independent experiments were performed.

### 2.4 Western blot

Cells were collected and lysed in RIPA buffer supplemented with 1 mM protease inhibitor PMSF to prepare total cell lysates. After detecting the protein concentration using the bicinchoninic acid (BCA) method, protein samples were separated by SDS-PAGE gels and transferred to polyvinylidene fluoride (PVDF) membranes. Then, PVDF membranes were blocked with 5% skim milk for 120 min at room temperature (RT) or overnight at 4 °C, incubated with primary and secondary antibodies, and finally developed with chemiluminescent substrates. The protein bands were detected on Tanon 5200 Multi using ECL Kit and quantified by Image J analysis.

### 2.5 RNA extraction and quantitative real-time PCR

Total RNA was extracted with TRIzol reagent (Tiangen, DP424) after discarding the supernatant of the cell culture and washing the cells twice with PBS, then subjected to reverse transcription with HiScript III RT SuperMix for qPCR (Vazyme, R323-01). PCR were performed with ChamQ Universal SYBR qPCR Master Mix (Vazyme, Q711) using ABI QuantStudioTM 3 (Applied Biosystems, CA, The United States). Relative expressions of specific genes were calculated by 2^−ΔΔCt^ method by normalizing to the house-keeping gene (β-actin) expression. Primers were indicated in **Table 1** and synthesized by Sangon Biotech (Shanghai, Co, Ltd.).

**Table 1:** Primers used in this study.

Target genes	Primer sequences (5′–3′)	Genbank accession no.	Species
IFN-β	F: CTCCACCACAGCTCTTTCCAT	NM_001003923	Sus scrofa
	R: TTGAGGAGTCCCAGGCAACT		
IFIT1	F: TCCGACACGCAGTCAAGTTT	NM_001548.5	Sus scrofa
	R: TGTAGCAAAGCCCTGTCTGG		
ISG15	F: GGCAGCACAGTCCTGTTGATGG	NM_001244363	Sus scrofa
	R: TGCGTCAGCCAGACCTCATAGG		
β-Actin	F: CTCCATCATGAAGTGCGACGT	XM_021086047	Sus scrofa
	R: GTGATCTCCTTCTGCATCCTGTC		

### 2.6 Immunofluorescence staining

Immunofluorescence staining cells were infected with PDCoV for designated time periods, then fixed in 4% paraformaldehyde for 10 min, permeabilized with 0.5% Triton X-100 in PBS for 10 min, blocked with 3% bovine serum albumin in PBS for 1 h at room temperature or 30 min at 37°C, and incubated with primary antibodies at 4 °C overnight. After washing with PBS, cells were incubated with secondary body at 37°C for 1 h, followed by staining with DAPI for 7 min at room temperature. Finally, the slides were observed under a confocal microscope (ZEISS, LSM 880NLO) with a 100× oil immersion objective. The images were analyzed using ZEN software.

### 2.7 Viral titration

Cells were seeded in 96-well plates with Dulbecco’s modified Eagle medium containing 10% fetal bovine serum and 100 U/mL penicillin/0.1 mg/mL streptomycin at 37 °C with 5% CO_2_ until grow into a single layer then incubated with diluted samples containing 5µg/mL Trypsin and 0.03% Tryptose Phosphate Broth at 37 °C with 5% CO_2_. After 72 h of incubation, the Reed and Muench method was used to calculate 50% tissue culture infective dose titer (TCID_50_).

### 2.8 Statistical analysis

The results are expressed as the mean ± Standard Deviation (SD) values. Significant differences among groups were determined with one-way analysis of variance followed by a Tukey test using statistical software GraphPad Prism. Values of *P < 0.05, **P < 0.01, ***P < 0.001 and ****P < 0.0001 were considered significant, and ns indicates a lack of significant difference.

## 3 Results

### 3.1 PDCoV infection induces mitochondrial damage

**Figure 1:**
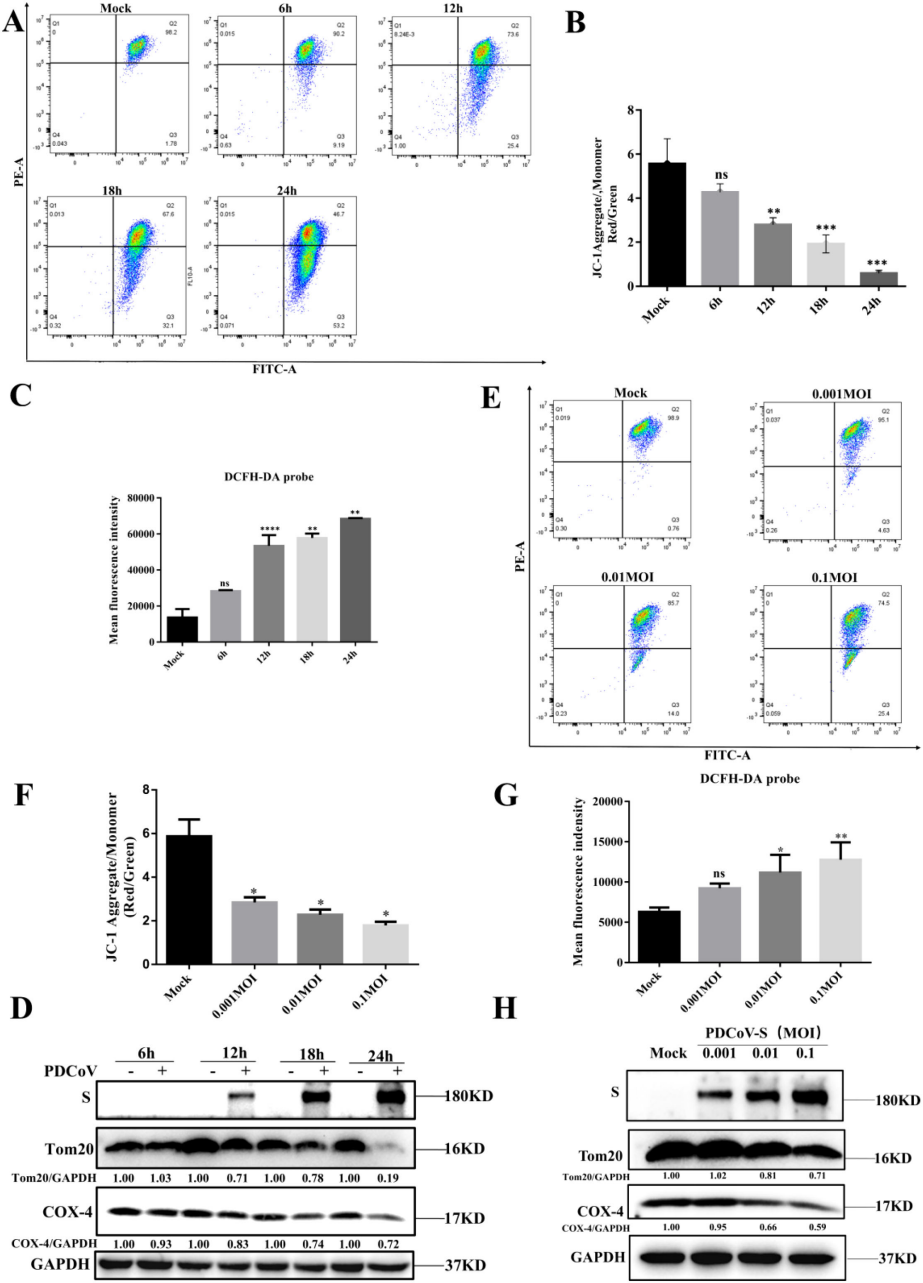
PDCoV infection leads to mitochondrial damage in a time- and dose-dependent manner in LLC-PK1 cells. **(A)** MMP was detected by flow cytometry using the JC-1 probe upon PDCoV (MOI = 0.1) infection LLC-PK1 cells at 6, 12, 18 and 24 hpi, 10000 events were gated per sample. **(B)** Quantification of mitochondrial membrane potential evaluated by the red/green ratio of JC-1 upon PDCoV (MOI = 0.1) infection LLC-PK1 cells at 6, 12, 18 and 24 hpi, 10000 events were gated per sample. **(C)** ROS was detected by flow cytometry using DCFH-DA probe at 6, 12, 18 and 24 hpi, and mean fluorescence intensity was measured. **(D)** Tom20 and COX-4 protein levels were determined by Western blot upon PDCoV infection (MOI = 0.1) at 6, 12, 18, and 24 hpi. The viral S protein was used as a marker for PDCoV infection. **(E)** MMP was detected by flow cytometry using the JC-1 probe upon PDCoV (MOI = 0.001,0.01,0.1) infection at 18 hpi. 10000 events were gated per sample. **(F)** Quantification of mitochondrial membrane potential evaluated by the red/green ratio of JC-1 upon PDCoV (MOI = 0.001,0.01,0.1) infection at 18 hpi, 10000 events were gated per sample. **(G)** ROS was detected by flow cytometry using DCFH-DA probe upon PDCoV (MOI = 0.001,0.01,0.1) infection at 18 hpi, and mean fluorescence intensity was measured. **(H)** Tom20 and COX-4 protein levels were determined by Western blot upon PDCoV (MOI = 0.001,0.01,0.1) infection at 18 hpi. The viral S protein was used as a marker for PDCoV infection. **, and *** indicate statistically significant differences with *P* < 0.01, and *P* < 0.001, respectively. ns indicates not statistically significant. All data are expressed as mean ± Standard Deviation (SD) of three independent experiments (n = 3).

Previous reports indicated that PDCoV infection can induce cellular ultrastructural changes, such as the presence of many cytoplasmic vesicles including dilated rough endoplasmic reticulum, Double Membrane Vesicles (DMVs) and injured mitochondria (3). To confirm the effects of PDCoV infection on mitochondrial morphology and structure in LLC-PK1 cells, JC-1 probes were utilized to evaluate the degree of mitochondrial dysfunction. After PDCoV infection, MMP was significantly reduced in a time-dependent manner (**Figures 1A, B**). Correlating with mitochondrial dysfunction, PDCoV infection significantly increased ROS levels in a time-dependent manner (**Figure 1C**). Furthermore, we assessed changes in mitochondrial protein (Tom20, translocase of outer membrane 20; COX-4, cytochrome C). As show in **Figure 1D**, mitochondrial protein levels all decreased in a time-dependent manner. Similarly, the above results also shown in a dose-dependent manner (**Figures 1F-H**). Therefore, the above results show that PDCoV infection results in mitochondrial damage.

### 3.2 PDCoV infection induces mitophagy

**Figure 2:**
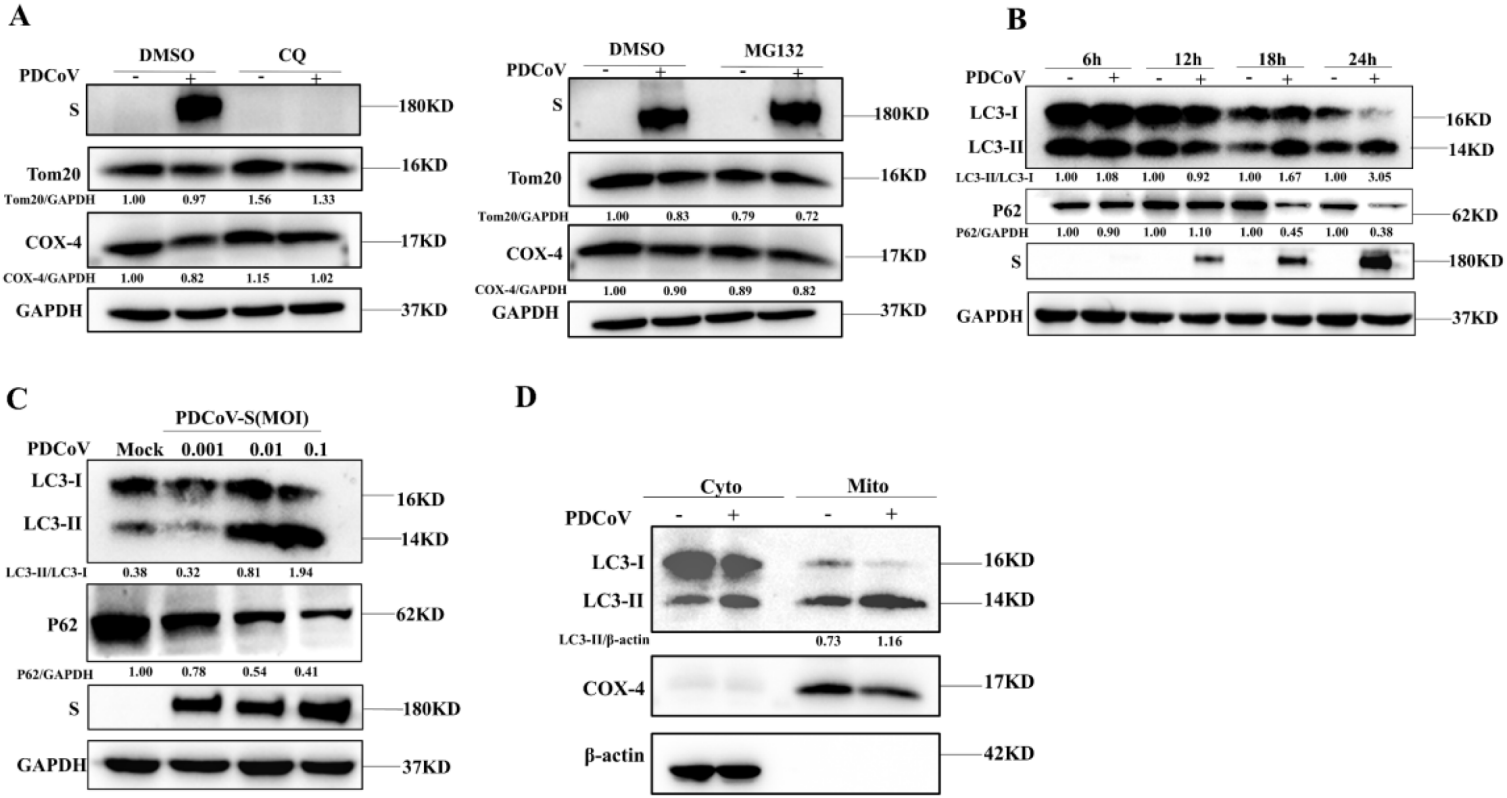
PDCoV infection induced mitochondria to be encapsulated by autophagosomes in LLC- PK1 cells. **(A)** PDCoV-S protein, Tom20 and COX-4 were determined by Western blot (MOI = 0.1) infection at 18 hpi in the presence of CQ (25 µM), MG132 (10 µM) treatment (Add CQ to the maintenance medium during medium change after incubating cells with PDCoV). **(B)** p62, LC3, and S proteins were determined by Western blot upon PDCoV (MOI = 0.1) infection at 6, 12, 18 and 24 hpi. **(C)** p62, LC3, and S proteins were determined by Western blot upon PDCoV (MOI = 0.001, 0.01, 0.1) infection at 18 hpi. **(D)** LC3 protein in mitochondrial lysates was measured by Western blot upon PDCoV infection (MOI = 0.1) at 18 hpi. All data are expressed as mean ± Standard Deviation (SD) of three independent experiments (n = 3).

**Figure 3:**
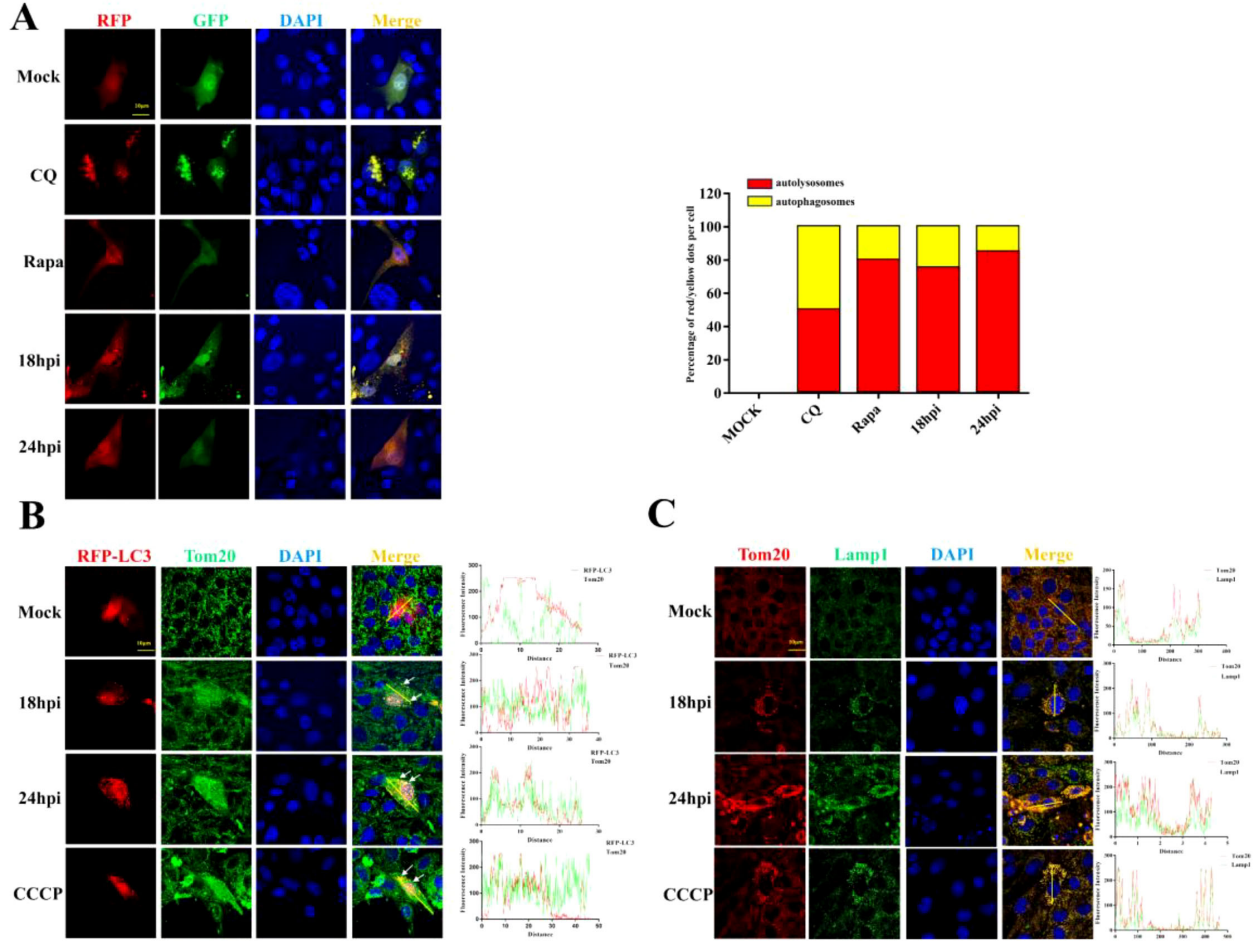
PDCoV infection triggers mitophagy in LLC-PK1 cells. **(A)** Cells transfected with RFP-GFP-LC3 plasmid were infected with PDCoV (MOI = 0.1) for 18 and 24 h or treated with CQ (25 µM) or Rapa (25 nM) for 24 h, and then observed via a confocal microscopy. Quantification of autophagic flux evaluated by the ratios of autolysosomes vs autophagosomes (Add Rapa to the maintenance medium during medium change after incubating cells with PDCoV). **(B)** Colocalization of mitochondria (Tom20, Green) with autophagosomes (LC3, red) was visualized by confocal microscopy upon PDCoV infection (MOI = 0.1) at 18 and 24 hpi or 10 µM CCCP treatment for 24 (h) **(C)** Colocalization of mitochondria (Tom20, Red) with lysosomes (Lamp1, green) was visualized by confocal microscopy upon PDCoV infection (MOI = 0.1) at 18 and 24 hpi or 10 µM CCCP treatment for 24 (h) Scale bars: 10 µm. All data are expressed as mean ± Standard Deviation (SD) of three independent experiments (n = 3).

Damaged mitochondria will be cleared by selective autophagy, referred to as mitophagy. In order to verify whether the depletion of mitochondria is associated with mitophagy upon PDCoV infection, we utilized chloroquine (an inhibitor of lysosomal acidification) to impair lysosomal degradation and then assessed the changes in mitochondrial numbers. As shown in **Figure 2A**, decreased Tom20 and COX-4 protein levels in PDCoV-infected cells were restored by CQ but not by the proteasome inhibitor MG132 (proteasome inhibitor), suggesting that PDCoV infection may induce mitophagy to clear the damaged mitochondria. Next, we determined the effects of PDCoV infection on autophagy by detecting protein levels of p62 and LC3-II protein, two classical autophagy protein markers. As shown by **Figure 2B**, PDCoV infection decreased p62 but increased LC3-II protein levels at 18 and 24 hpi significantly. Moreover, PDCoV infection decreased p62 but increased LC3-II protein levels at 18 hpi in dose-dependent (**Figure 2C**). Furthermore, immunoblot assays showed that the expression of LC3-II was increased in mitochondrial lysates (**Figure 2D**). RFP-GFP-LC3 plasmid was transfected and the level of LC3-II protein in mitochondria was used to perform an autophagic flux assay. PDCoV infection significantly induced the formation of autolysosomes at 18 and 24 hpi, similar as observed when adding rapamycin (**Figure 3A**). These results indicated that PDCoV infection induced completely autophagy. As shown in **Figures 3B, C**, PDCoV infection not only increased co-localization between mitochondria (Tom20 labeled) and autophagosomes (RFP-LC3, LC3 labeled), but also between mitochondria (Tom20 labeled) and lysosomes (Lamp1 labeled) at 18 and 24 hpi. Taken together, it can be concluded that PDCoV infection induces mitophagy to clear impaired mitochondria at late stages of viral infection.

### 3.3 Mitophagy inhibits IFN-I response to promote PDCoV replication

**Figure 4:**
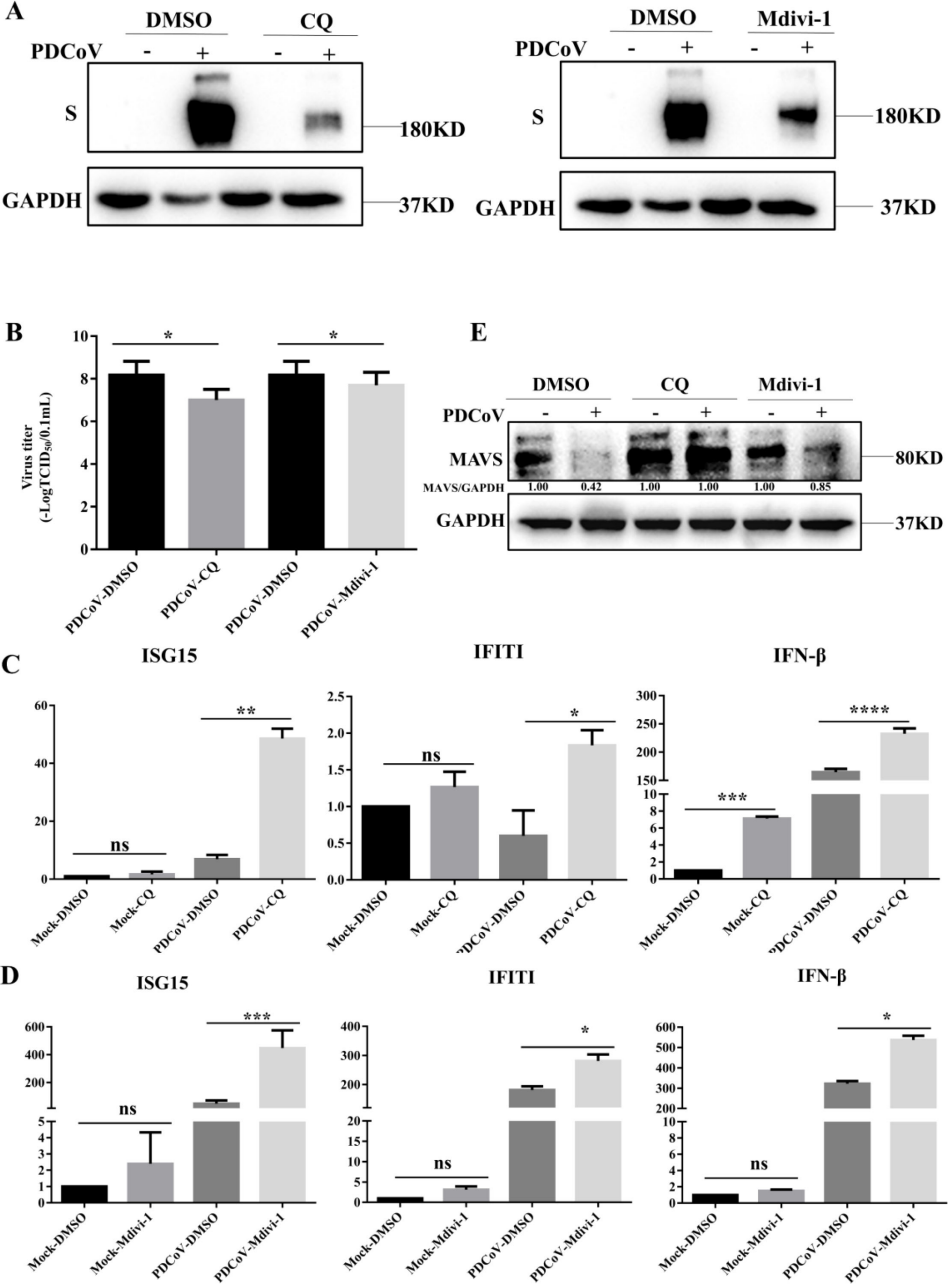
Mitophagy promotes PDCoV replication in LLC-PK1 cells. **(A)** Viral S protein levels were determined by Western blot. **(B)** Supernatants were collected to determine viral titers measured by TCID_50_ assay. Cells were infected with PDCoV in the absence or presence of Mdivi-1 (25 µM) for 18h (Add Mdivi-1 to the maintenance medium during medium change after incubating cells with PDCoV). **(C)** IFN-β, IFIT1, ISG15 mRNA levels were determined by q-PCR upon PDCoV infection for 18 h in the absence or presence of CQ. **(D)** IFN-β, IFIT1, ISG15 mRNA levels were determined by q-PCR upon PDCoV infection for 18 h in the absence or presence of Mdivi-1. **(E)** MAVS protein levels were determined by Western blot upon PDCoV infection (MOI = 0.1) at 18hpi. *, **, *** and **** indicate statistically significant differences with *P* < 0.05, *P* < 0.01, *P* < 0.001 and *P* < 0.0001, respectively. ns indicates not statistically significant. All data are expressed as mean ± Standard Deviation (SD) of three independent experiments (n = 3).

To determine the role of mitophagy in PDCoV replication, mitochondrial division inhibitor (Mdivi-1), which blocks mitochondrial fission to inhibit mitophagy, and the lysosomal acidification inhibitor CQ, were utilized. As shown in **Figure 4A**, the expression of S protein in PDCoV-infected cells was suppressed by addition of CQ or Mdivi-1. In line with this, PDCoV titers in culture supernatant were decreased by the addition of either CQ or Mdivi-1(**Figure 4B**). To explore whether the impact of mitophagy on PDCoV replication may correlate with an impact on the antiviral type I interferon system, activation of IFN signaling proteins and mRNA expression of IFN-β and the IFN-stimulated genes tetratricopeptide repeats 1 (IFIT1) and ISG15 were evaluated. Inhibition of PDCoV-induced mitophagy via addition of CQ, Mdivi-1 resulted in increased expression of IFN-β, IFIT1 and ISG15 (**Figures 4C, D**). MAVS is a key antiviral protein located in the mitochondria and the hub protein of the RIG-I signaling pathway that leads to IFN production. PDCoV infection resulted in the reduced levels of MAVS protein (**Figure 4E**). The reduced MAVS protein could be restored by the inhibition of PDCoV-induced mitophagy via addition of CQ, Mdivi-1. Taken together, PDCoV activates mitophagy to suppress antiviral IFN-I system and enhance virus replication, which possibly related to mitophagy degradation of MAVS.

## 4 Discussion

As a strictly cell-dependent parasites, coronaviruses play chess game with the cell all the way (21, 22). Previous studies indicate that NBR1 can restrict PDCoV replication by selective autophagy degradation of viral E protein, while simultaneously demonstrating how PDCoV counteracts this defense through NSP5-mediated cleavage of NBR1 (23). The invasion of virus causes damage to the mitochondria. Subsequently, the infected cells can promptly remove the fragmented mitochondria by autophagy to maintain normal metabolism (10, 24, 25). Meanwhile, the virus utilizes mitophagy to facilitate its own replication. Li et al. demonstrated that M protein of coronavirus promotes mitophagy over virophagy by recruiting PDPK1 to phosphorylate SQSTM1 at T138 (26). In this study, we revealed that PDCoV infection can induce mitophagy to promote MAVS degradation and enhance viral replication.

Mitophagy is a process of eliminating fragmented mitochondria through autophagy, thereby adjusting the number of mitochondria and maintaining the stability of cellular metabolism (27–29). A previous study revealed that the organelle membranes can be rearrangement after infected of PDCoV and trigger autophagy (3). Meanwhile, Yang et al. demonstrated that PGAM5 can degrade N protein of PDCoV via the autophagy pathway and activate type I interferon to antagonize viral replication (30). Moreover, M protein of coronavirus can promote mitophagy by recruiting PDPK1 to phosphorylate SQSTM1 at T138 (26), ORF10 of SARS-CoV-2 can suppress the antiviral innate immune response by degrading MAVS through mitophagy, and NSP8 of SARS-CoV-2 can also induce mitophagy by damaging mitochondria (17, 18). Consistent with these findings, our study demonstrated that PDCoV infection induces mitophagy in LLC-PK1 cells, caused damage to the structure and function of mitochondria at 18-24h after PDCoV infection by the evidence of significantly decreased MMP and increased ROS. Meanwhile, the number of mitochondria decreased significantly (**Figure 1**). Furthermore, PDCoV triggers mitophagy to suppress the production of IFN I by degrading MAVS (**Figure 4**), revealed that PDCoV infection triggers mitophagy to escape host innate immunity. This is in line with results on SARS-CoV-2 ORF10 and PEDV Nsp14 infected induces mitophagy, which also showed virus can suppress the antiviral innate immune response by degrading MAVS through mitophagy (26, 31).

One limitation in this study is that we did not identify which pathway to induce mitophagy. In this study, we found that PDCoV infection downregulated the expression of FUNDC1 (FUN14 domain containing 1) and TOLLIP (Toll-Interacting Protein), indicating that PDCoV infection does not trigger these receptors mediated mitophagy. In addition, we detected the Parkin levels in mitochondria of PDCoV infected cells. The results showed that the protein level of Parkin did not change (**Supplementary Figure S1**), indicating that PDCoV infection does not trigger PINK1-Parkin pathway mediated mitophagy. BCL2 Interacting Protein 3/NIP3-like protein X (BNIP3/NIX) are mitophagy receptors located on the outer mitochondrial membrane, which can directly bind to LC3 and trigger mitophagy (20, 29). In addition, autophagy and beclin 1 regulator 1 (AMBRA1, an autophagy core complex component) and optineurin (OPTN, a mitophagy adaptor protein) both regulate mitophagy (32, 33). However, whether these receptors can trigger the mitophagy pathway in PDCoV-infected LLC-PK1 cells remain unknown. The auxiliary protein NS7 of PDCoV is previously reported have a definite co-localization with mitochondria. However, there have no reports related to its association with mitophagy yet (34). Moreover, another study reported that NSP15 protein of PDCoV antagonizes interferon-β production independently of its endoribonuclease activity (9). Therefore, we transfected NS7 or NSP15 plasmids into LLC-PK1 cells to investigate which protein mediate mitophagy. Unfortunately, these two proteins did not induce mitophagy by the results of unchanged ratio of LC3-II/LC3-I expression (data not shown). Therefore, studies on which protein(s) trigger mitophagy and degrade MAVS warrants further investigation.

To summarize, we demonstrate that PDCoV infection triggers mitophagy to inhibit the IFN I response and promote viral replication. Our findings enhance understanding of the important role of mitophagy in PDCoV replication and shed light on PDCoV resistant of antiviral innate immunity by degradation MAVS, which may lead to new strategies for antiviral therapy.

## Data Availability

The original contributions presented in the study are included in the article/**Supplementary Material**. Further inquiries can be directed to the corresponding author.

## Supplementary material

Supplementary Figure 1 The role of TOLLIP/FUNDC1/Parkin-PINK1 in triggering mitophagy after PDCOV infects LLC - PK1 cells. **(A)** PDCoV-S protein, TOLLIP and FUNDC1 were determined by Western blot (MOI = 0.1) at 6h, 12h, 18h, 24h. **(B)** PDCoV-S protein, PINK1/P-PINK1 and Parkin/P-PINK1 were determined by Western blot (MOI = 0.001, 0.01, 0.1) at 18h. All data are expressed as mean ± Standard Deviation (SD) of three independent experiments (n = 3).
