# Supplementary material for: Porcine deltacoronavirus infection triggers mitophagy to dampen the interferon response and promote viral replication
Source: Front Immunol. 2025 Oct 13;16:1684178. doi: 10.3389/fimmu.2025.1684178 (PMC12554741; doi:10.3389/fimmu.2025.1684178)
Supplement: Supplementary Figure 1 — The role of TOLLIP/FUNDC1/Parkin-PINK1 in triggering mitophagy after PDCOV infects LLC - PK1 cells. (A) PDCoV-S protein, TOLLIP and FUNDC1 were determined by Western blot (MOI = 0.1) at 6h, 12h, 18h, 24h. (B) PDCoV-S protein, PINK1/P-PINK1 and Parkin/P-PINK1 were determined by Western blot (MOI = 0.001, 0.01, 0.1) at 18h. All data are expressed as mean ± Standard Deviation (SD) of three independent experiments (n = 3). [file DataSheet1.docx]

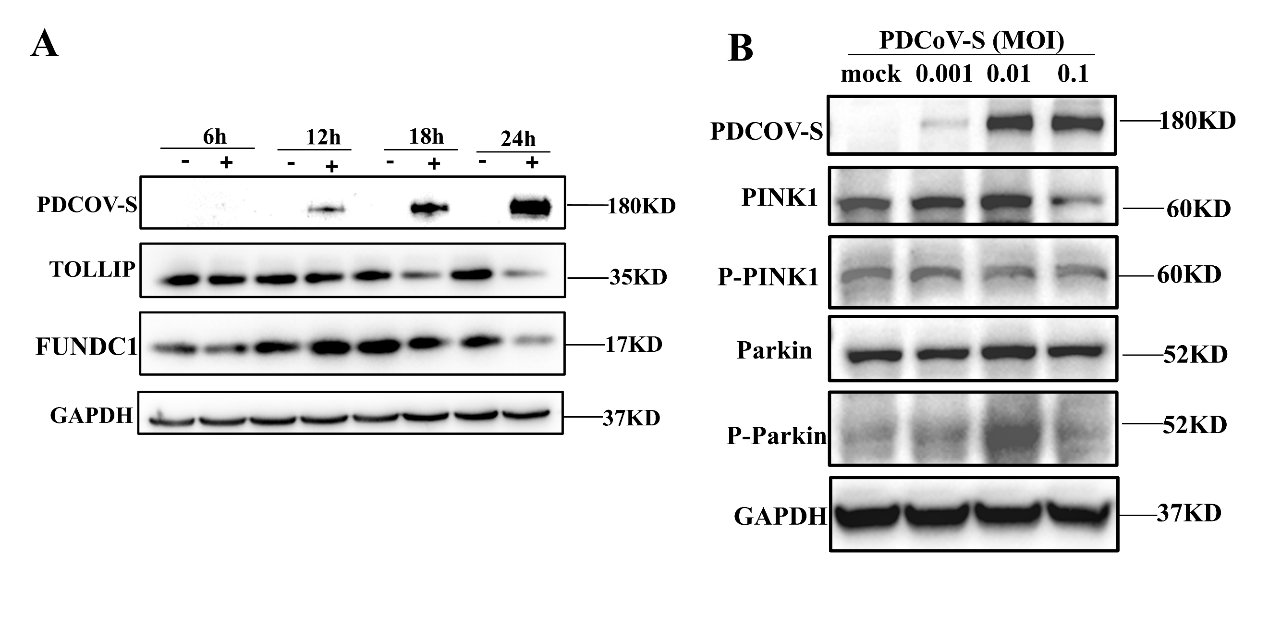


**FIGURE S1** The role of TOLLIP/FUNDC1/Parkin-PINK1 in triggering mitophagy after PDCOV infects LLC - PK1 cells. (A) PDCoV-S protein, TOLLIP and FUNDC1 were determined by Western blot (MOI = 0.1) at 6h, 12h, 18h, 24h. (B) PDCoV-S protein, PINK1/P-PINK1 and Parkin/P-PINK1 were determined by Western blot (MOI = 0.001, 0.01, 0.1) at 18h. All data are expressed as mean ± Standard Deviation (SD) of three independent experiments (n = 3).
